# First Case of Clinical Cat Aelurostrongylosis in the Brazilian Amazon: Clinical and Molecular Insights

**DOI:** 10.3390/pathogens10050595

**Published:** 2021-05-13

**Authors:** Wilison da Silva Lima, Enny Caroline Ferreira Farago, Millena do Nascimento Mesquita, Acácio Duarte Pacheco, Patrícia Fernandes Nunes da Silva Malavazi, Hugo Salvador Oliveira, Simone Morelli, Mariasole Colombo, Angela Di Cesare, Soraia Figueiredo de Souza

**Affiliations:** 1 Centro de Ciencias Biológicas e da Natureza, Federal University of Acre, Rio Branco, AC 69900-000, Brazil; wilsonsilvalima@gmail.com (W.d.S.L.); alloraenny@hotmail.com (E.C.F.F.); mesquitamillena@hotmail.com (M.d.N.M.); acacio.pacheco@ufac.br (A.D.P.); patricia.malavazi@ufac.br (P.F.N.d.S.M.); soraiasouza@yahoo.com (S.F.d.S.); 2 Autonomous Veterinarian, Indaiatuba, SP 13330-000, Brazil; hugo@veterinario.med.br; 3 Faculty of Veterinary Medicine, University of Teramo, Località Piano D’Accio, 64100 Teramo, Italy; mcolombo@unite.it (M.C.); adicesare@unite.it (A.D.C.)

**Keywords:** cats, *Aelurostrongylus abstrusus*, Brazil, molecular, Baermann

## Abstract

*Aelurostrongylus abstrusus* is the most important respiratory parasite infecting domestic cats worldwide. Nevertheless, most records and epizootiological data come from Europe, whilst poor and fragmentary information are available for other regions, including the Americas. The present article describes the first description of cat aelurostrongylosis from Amazonia, Brazil. Eighty-one cats, 13 from a shelter and 68 admitted at the Teaching and Research Unit in Veterinary Medicine (UV) at the Federal University of Acre (UFAC), Brazil, were included in the study. For all cats, three faecal samples from consecutive defecations were examined using the Baermann’s technique. Nematode first stage larvae (L1), retrieved in 2/81 (2.5%) samples, were microscopically identified as *A. abstrusus* and then subjected to a molecular assay able to identify the three most important species of metastrongyloids infecting felids. This test confirmed the *A. abstrusus* identity in one sample, while the second scored negative. The cat with confirmed aelurostrongylosis showed radiographic changes, i.e., an interstitial pattern, compatible with the infection. The other cat, which scored positive at the Baermann’s examination, was apparently healthy at the physical examination and showed no thoracic alterations. The occurrence of *A. abstrusus* in domestic cats from Brazilian Amazon is herein demonstrated for the first time. Clinical, epizootiological and molecular implications are discussed.

## 1. Introduction

Respiratory metastrongyloids are nowadays ranked as primary parasites in feline medicine. Metastrongyloids include different species of nematodes, being the angiostrongylid *Aelurostrongylus abstrusus* and the crenosomatid *Troglostrongylus brevior* the most frequent in domestic cats and wild felines [1,2]. *Aelurostrongylus abstrusus* is the so-called “cat lungworm”. It has a worldwide distribution and is the most important respiratory parasite of domestic cats (*Felis catus*) [2]. Though rarely, wild felids may also act as definitive hosts for this metastrongyloid, although its real prevalence in wildlife is yet to be elucidated [2]. Adults of *A. abstrusus* reside in nodules in the bronchioles, alveolar ducts and alveoli of infected definitive hosts [1]. Females produce eggs that hatch within the parenchyma and release first stage larvae (L1) which reach the pharynx via the muco-ciliary escalator and are then swallowed and passed to the environment with the faeces [1]. The life cycle is indirect and L1 develop to the third (infective) larval stage (L3) inside different species of terrestrial gastropods (i.e., slugs or snails) that act as intermediate hosts [1,3,4,5]. However, cats usually become infected ingesting paratenic hosts such as rodents, amphibians, birds and small reptiles [2]. Cat aelurostrongylosis may be either subclinical or course with respiratory clinical signs such as cough, dyspnea, tachypnea, open-mouth breathing [1,6]. In severe cases, lung damages may lead to pulmonary hypertension, with potential fatal consequences [7,8]. Currently, the copromicroscopic detection of L1 with the Baermann’s method is the gold standard for diagnosing cat aelurostrongylosis in clinical settings. However, DNA-based approaches overcoming the limitations inherent to the Baermann’s technique are pivotal for the molecular confirmation of the identity of L1 isolated at the faecal examinations [2]. Novel serological techniques are also being increasingly used in epizootiological settings [9,10]. 

Over time, aelurostrongylosis has been recorded mostly from Europe in both domestic [9,10,11,12,13,14,15] and wild [16,17,18] felines. Epizootiological information from other territories are patchy and limited, though the presence of the parasite is known for instance, in South Africa [19], Australia [20] and North [21,22] and Insular Americas [23]. Fragmentary information is available on the distribution of *A. abstrusus* in South America, where very recently this nematode has been declared to be neglected and underestimated [24]. Accordingly, knowledge on *A. abstrusus* in Brazil is very poor probably because this nematode is factually overlooked, though a documented presence in definitive and intermediate hosts [24,25,26,27,28,29,30]. No data are known on its occurrence in felines living in the Brazilian Amazonas, albeit its recent detection in gastropod intermediate hosts. For instance, in a recent study carried out in Rio Branco, Acre, an infection rate ~22% by *A. abstrusus* was described in the giant African snail *Lissachatina fulica* [31]. The present study reports for the first time the occurrence of *A. abstrusus* in domestic cats living in Brazilian Amazonia, to update knowledge on this lungworm in South America and provide clinical and molecular insights.

## 2. Results

### 2.1. Infection Rate 

Two out of the 81 cats (2.5%) were positive for metastrongyloid L1 at the Baermann’s test. The larvae were microscopically identified as *A. abstrusus* and the identity was molecularly confirmed in one of the two samples. 

The sequencing of the amplicon produced a sequence (GenBank accession number MZ093629) with <100% homology with all *A. abstrusus* sequences deposited in the GenBank database and the closest homology with an isolate from Italy (Accession Number 372965.2, homology 98.85% due to six nucleotidic substitutions). The second sample scored negative to the genetic test performed three times. 

### 2.2. Clinical Findings

**Figure 1:**
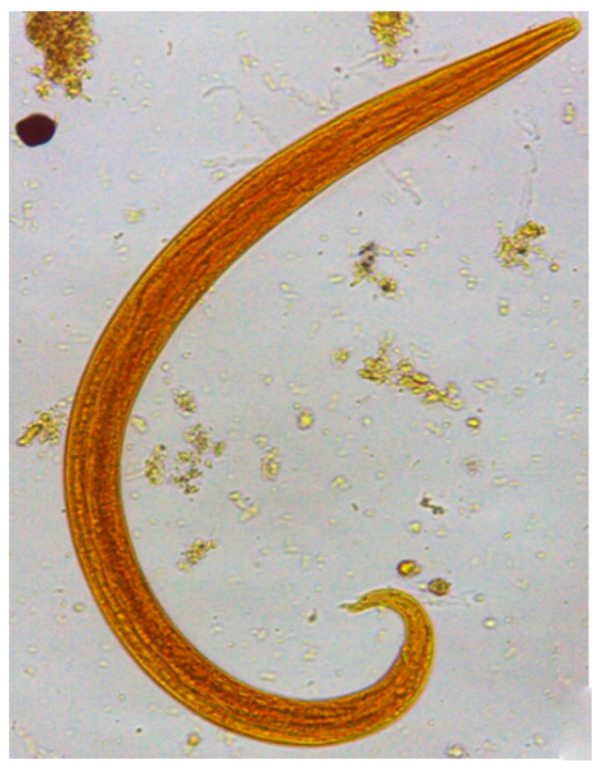
First stage larva of *Aelurostrongylus abstrusus* retrieved at the Baermann examination in the faeces of a cat from the municipality of Rio Branco, Acre, Brazil. 40× magnification.

The animal (Case 1) diagnosed with aelurostrongylosis upon microscopy (Figure 1) and PCR was a six-month old male cat, adopted when stray and ageing less than 30 days. The cat was vaccinated but had never been dewormed. The animal was housed indoors but with access to the yard of the house. At the clinical examination, the cat displayed a marked tachypnea (240 respiratory movements per minute) with no other evident clinical abnormalities. 

**Figure 2:**
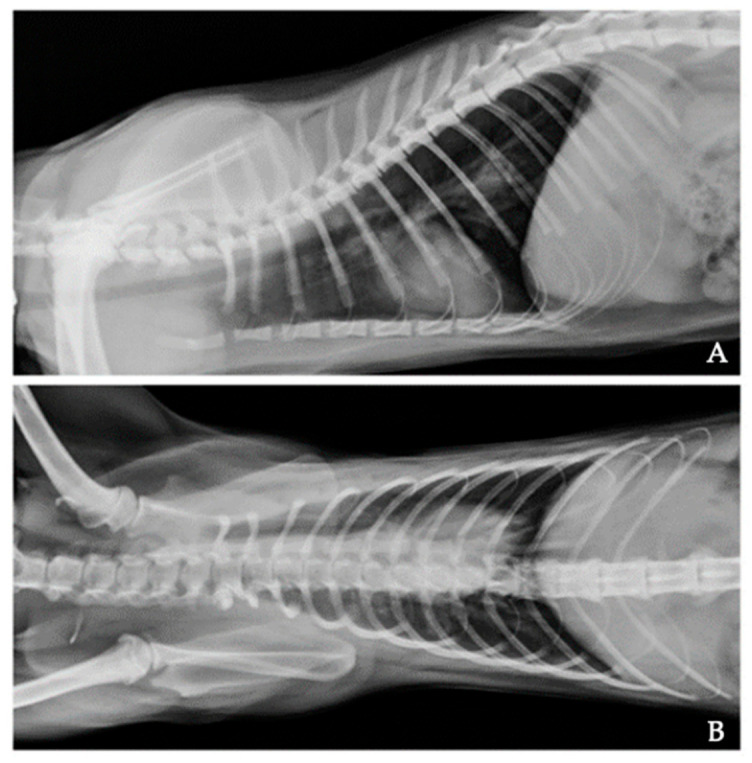
Latero-lateral (**A**) and ventro-dorsal (**B**) projections of thoracic radiographs of the cat diagnosed with aelurostrongylosis showing an interstitial pattern with increased radiopacity in the perihilar region and caudal portion.

After the microscopic diagnosis of aelurostrongylosis, complete blood count and biochemical profile were performed to assess the general health condition of the animal, and no alterations were detected. Thoracic radiographs showed an interstitial pattern (Figure 2), especially in the perihilar region and caudal lobes of the lung. During the radiographic examination, performed with no sedation or anaesthesia, the cat presented an episode of dyspnoea.

The cat weighted 1.7 kg and the responsible veterinarian recommended a treatment regimen of oral fenbendazole 50 mg/kg daily for five consecutive days. The owner referred again the cat for a parasitological re-evaluation after 120 days and informed the veterinarian that the animal cat received a single dose of fenbendazole. At the Baermann’s test, *A. abstrusus* larvae were again detected, thus a second cycle of treatment with five days of fenbendazole 50 mg/kg was recommended again. No further follow-ups were performed.

The second cat (Case 2) positive at the faecal examination (but not at the PCR) was a 1-year-old female from an animal shelter in the city. It had no history of vaccination and had been dewormed with 50 mg of mebendazole about three months prior to the present study. During the physical examination, the cat was eupnoeic, with no changes in cardiopulmonary auscultation. A single larva microscopically identified as *A. abstrusus* L1 was found at the Baermann’s test. Thoracic X-rays were performed also on this animal, but no evident alterations were observed. The treatment scheme used for the previous cat was applied also in this case. No further clinical procedures nor follow-ups were possible due to lack of compliance of the shelter caretaker.

## 3. Discussion

This is the first molecularly confirmed *A. abstrusus* infection in domestic cats from Brazil and the first report of cat aelurostrongylosis in Brazilian Amazonia. Thus, the enzooticity of *A. abstrusus* in Brazil is herein confirmed as previously suggested by past [32] and recent descriptions of gastropods (*L. fulica*) naturally harbouring larvae of *A. abstrusus* in the Amazonia [31]. The infection rate here detected appears low if compared for instance to the rates described in Europe, e.g., 6.6% in Germany [33], 10.3% in Italy [12], 8–13.2% in Greece [10,34], 14.5% in Hungary [35],17.4% in Portugal [36], but compatible with values published for the Americas, i.e., 2.1% in USA [37], 0.4% in Colombia [38].

Previous studies from South America reported prevalence rates of 0.21% in Colombia, 8.6% in Uruguay, from 2.6 to 35.3% in Argentina and from 1.3 to 29.5% in Brazil [24]. Literature data on the occurrence of *A. abstrusus* in Brazil from the last two decades describe infection rates of 1.37–18% [27,39,40,41,42,43,44], thus the herein results are in line with these lower range values. However, it should be taken into account that a relatively small number of cats has been examined in the present study, and this is not representative of the territorial epizootiological scenario. Thus, the factual occurrence of *A. abstrusus* in cat populations of Brazil and of local Amazonia could be likely underestimated. This is particularly true also because cats living in the study area are not regularly checked for parasites nor treated with parasiticides (personal observations of the authors). Altogether, it can be argued that a true underestimation of the overall prevalence of aelurostrongylosis in populations of domestic cats from Amazonia and Brazil in general is realistic. As the scope of the present article is to describe the first molecularly confirmed infection by enzootic *A. abstrusus* in cats of this region and not to perform an extensive epizootiological study, further focused studies are thus warranted. As epizootiological knowledge on feline lungworms in South America is still in its infancy and detailed data are lacking, it is difficult to draw any comparison between information from South America with those available from Europe, where epizootiological surveys focusing on feline lungworms have been very frequently conducted in the past years [10,12,15]. It is worthy of note that most reports of aelurostrongylosis in Brazil are published in local Journals and molecular confirmations on the identity of *A. abstrusus* isolated from domestic cats were never provided. The possibility that some past records of cat aelurostrongylosis without molecular confirmation of larval identity were misdiagnosed with other nematodes cannot be ruled out [2].

While diagnostic molecular tools are commonly used elsewhere for the unequivocal identification of lungworms infecting felids in both single clinical cases and epizootiological studies in intermediate and definitive hosts [4,5,8,10,15,45,46,47,48], they are less frequently used in South America due to the relatively high costs of these procedures and equipment in certain settings [24]. The morphological and morphometrical evaluation of L1 retrieved at the Baermann’s test require a well-trained expert operator and can be insufficient for a definitive identification to species of cardio-respiratory metastrongyloids affecting felines, i.e., *A. abstrusus*, *Troglostrongylus* spp., *Oslerus rostratus* and *Angiostrongylus* spp., as their larvae may present overlapping features [2,22,23]. This is particularly true for areas where bridging infections and parasite-sharing is favoured, i.e., where wild felids and domestic cats live in sympatry or have overlapping distribution areas and may be infected by more than one lungworm [2,15,47]. Under these circumstances, PCR-based tools are important to ultimately confirm infections in domestic cats, as in the case here presented. The implementation of genetic assay in areas of South America, as wild Amazonia, would also be important to provide accurate information on the occurrence of cardiorespiratory nematodes in wild felids and estimate possible transmission patterns and risk to domestic cats. For instance, previous *A. abstrusus* reports in wild felids from South America are ambiguous due to the lack of molecular analysis and can be attributable also to other lungworms [2]. A genetic identification of the parasites found in those studies would have provided important data. Molecular surveys in these regions would allow to understand which metastrongyloids nematodes, known or yet undescribed, may affect wildlife and domestic cats living in remote and wild territories geographies. In this view, it is worthy of note that a new species of metastrongyloid, i.e., *Angiostrongylus felineus*, has been recently described in a jaguaroundi in Brazil [49]. However, this apparent novel species has been thoroughly described microscopically but no molecular data have been generated, thus rendering impossible to state with certainty that it is a valid species, and it remains to elucidate if it could be a morphotype of other *Angiostrongylus* spp., e.g., *Angiostrongylus chabaudi*, i.e., a related species which affect felids in Europe, and to define geographical and host ranges. Data indicate that feline angiostrongylosis has a negligible importance in domestic cats of Europe [45,50] and, analogously, the use of molecular techniques would be powerful to understand what is the impact of *A. felineus* in felids of South America. Indeed, studies on elusive felids living in wild and remote areas are difficult, but further studies are truly encouraged. In this view, the successful recent use of molecular methods to obtain information on the occurrence of metastrongyloids in intermediate hosts of South America are very encouraging [3,31].

Data available on the clinical impact of respiratory nematodes of cats in Brazil are also few. Clinical evidence of Case 1 is compatible with aelurostrongylosis, as also the described radiographic alterations are common in cats infected by *A. abstrusus* and suggest an infection which has occurred weeks or months before the diagnosis. In fact, the interstitial pattern (Figure 2) is very common in cats infected by *A. abstrusus* and usually takes place after the onset of an acute phase which could be characterized by an alveolar pattern [6]. With regard to therapeutic options, the referring veterinarian has chosen a regime which is most often off label (i.e., formulations containing fenbendazole are licensed to treat aelurostrongylosis in a few countries). Indeed, off label treatment with oral fenbendazole, that is effective if administered for three consecutive days or more [51,52], was herein prescribed by the veterinarian probably due to its reduced costs if compared to other medication potentially effective in the treatment of *A. abstrusus* infections. In particular, the efficacy of macrocyclic lactones against feline metastrongyloids has been several times proved in pilot studies, case series and clinical trials for marketing authorizations [52,53,54,55,56]. Among these products, a topical spot-on product containing imidacloprid 10%/moxidectin 1%, is available in Brazil. Moreover, another marketed formulation containing milbemycine oxime (not labelled either in Europe) that showed a certain efficacy in stopping the larval shedding in single clinical cases [57], is available on the Brazilian market. Thus, their use could be considered by veterinarians when treating feline cardiopulmonary infections as an alternative to consecutive days protocols which, as in the present case, could cause lack of owner adherence to the recommendation of veterinarians. In this view, no conclusions can be drawn on the efficiency of the selected treatment in Case 1. In fact, consecutive days regimen of oral fenbendazole have indeed proven efficacious in some studies [51,52], but in this case the lack of owner compliance has likely caused a treatment failure explaining the persistence of the infection 4 months after dosing. On the other hand, considering the prepatent period of *A. abstrusus* (around 2 months), a re-infection, regardless of the persistence of the current infection, cannot be ruled out. 

The reasons why the cat of Case 2 scored negative at the PCR could be various. Given that only one larva was found at the faecal examination, it could be possible that this cat had a very low parasitic burden with a negligible larval shedding and thus, low amounts of DNA impairing its detection in the Baermann’s sediment. This hypothesis seems to be supported by the absence of any evident clinical signs or radiographic alterations. Another possibility for the negativity in the PCR is that the *A. abstrusus* isolate infecting the cat had some genetic mutation in the ITS2 region impairing the annealing of the species-specific primer used in the triplex PCR [58]. This possibility cannot be excluded considering that nucleotidic substitutions have been found in the isolate infecting Case 1 and no data are available on the genetic make-up of *A. abstrusus* populations occurring in South America where, as in Europe, this nematode has the potential to cause bridging infections between wild and domestic felids [2,24,59]. In fact, it has been recently shown that wildlife may act as a reservoir for genetic parasite recombination leading to high genetic variability of *Angiostrongylus vasorum*, i.e., another closely related angiostrongylid nematode. Thus, it cannot be ruled out that similar phenomena may occur in remote regions where parasites are shared between different animal species. As similar examples, in the last decade, novel strains of tick-borne agents of felids have been identified in Brazil [60] and in Europe [61,62]. Arthropod-borne diseases are highly enzootic and circulate between domestic and wild felids of Brazil and Europe, where they can also be shared with canids living in sympatry [60,61,63,64,65,66,67,68,69,70]. Therefore, further studies are necessary to investigate any genetic variability in *A. abstrusus* isolates which infect domestic cats and wildlife living in Amazonia and Brazil and if, as for other metastrongylid nematodes and arthropod-borne pathogens, there is a potential impact on their genetic make-up by bridging infections between different animal species. Other than an early phase of the infection and/or genetic mutations in the isolates, another possibility to take into account is that the Baermann’s test for Case 2 yielded a false positive result due to a laboratory contamination or inadvertent ingestion of the larva from the environment. 

In conclusion, regardless if the cat in Case 2 was truly infected or not, cat aelurostrongylosis is now regarded as enzootic in the Amazonia, where veterinarians need to be aware of the importance of this disease and copromicroscopic evaluation by the Baermann’s technique should be included as a routine examination in healthy cats and applied in all cats with respiratory signs. In fact, in this area the nematode occurs in mollusk intermediate hosts and domestic cats (present report), thus completing its biological cycle and causing clinical disease in cats. Additionally, cat owners living in enzootic areas must be conscious of the importance of deworming programs for their pets and understand the positive impact of adhering to the recommendations of their veterinarians. 

## 4. Materials and Methods

The present study was conducted at the Teaching and Research Unit of the Federal University of Acre campus Rio Branco (UV/UFAC). The study project was submitted to the Ethics Committee on the Use of Animals-CEAU/UFAC, and approved under number 23107.00856/2019-16. 

Faecal samples were collected from 81 cats aged > 2 months, i.e., 13 from an animal shelter and 68 admitted at UV/UFAC for routine checks or varying medical reasons, from June 2019 to January 2020. For each cat enrolled in the study, faecal samples were collected immediately after defecation from the soil or the litter box, for three consecutive days. Samples were stored in universal collectors, kept in a polystyrene box with ice and transported to the UFAC Clinical Analysis Laboratory, where they were processed using a conventional Baermann’s technique [71]. The identification of L1 was performed according to the morphological and morphometric characteristics of pulmonary metastrongyloid nematodes affecting felids [15,16]. Positive Baermann’s sediments were sent to the Faculty of Veterinary Medicine, University of Teramo, Italy to be subjected to a DNA-based assay, targeting the ITS2 region as the primary gene target, able to simultaneously discriminate and differentiate the three most important metastrongyloid nematodes affecting felids (i.e., *A. abstrusus*, *T. brevior* and *A. chabaudi*) in a range of biological samples [45,58,72]. Molecular analyses were carried out following procedures previously described and using the primers set NC1-NC2 for the first step, and the primers AabFor, TbrFor and AChFor in combination with NC2 for obtaining specific amplifications [58]. The amplicons were purified and sequenced, and sequences were determined in both directions. The electropherograms were verified by eye with the software Chromas Lite.

Clinical records and examinations were obtained from the cat which scored positive for respiratory nematodes. 

## Data Availability

All the data produced are reported in the article.
